# Removal of well-fixed components in femoral revision arthroplasty with controlled segmentation of the proximal femur

**DOI:** 10.1186/s13018-014-0137-9

**Published:** 2014-12-31

**Authors:** Panagiotis Megas, Christos S Georgiou, Andreas Panagopoulos, Antonis Kouzelis

**Affiliations:** Department of Adult Reconstructive Surgery, Patras University Hospital, Patras, Greece

**Keywords:** Proximal femur segmentation technique, Femoral osteotomy, Stem removal, Wagner revision stem, Hip revision arthroplasty, Surgical technique

## Abstract

**Background:**

The transfemoral and the extended trochanteric osteotomies are the most common osteotomies used in femoral revision, both when proximal or diaphyseal fixation of the new component has been decided. We present an alternative approach to the trochanteric osteotomies, most frequently used with distally fixated stems, to overcome their shortcomings of osteotomy migration and nonunion, but, most of all, the uncontrollable fragmentation of the femur.

**Methods:**

The procedure includes a complete circular femoral osteotomy just below the stem tip to prevent distal fracture propagation and a subsequent preplanned segmentation of the proximal femur for better exposure and fast removal of the old prosthesis. The bone fragments are reattached with cerclage wires to the revision prosthesis, which is safely anchored distally. A modified posterolateral approach is used, as the preservation of the continuity of the abductors, the greater trochanter, and the vastus lateralis is a prerequisite.

**Results:**

Between 2006 and 2012, 47 stems (33 women, 14 men, mean age 68 years, range 39–88 years) were revised using this technique. They were 12 (26%) stable and 35 (74%) loose prostheses and were all revised to tapered, fluted, grit-blasted stems. No fracture of the trochanters or the distal femur occurred intraoperatively. Mean follow-up was 28 months (range 6–70 months). No case of trochanteric migration or nonunion of the osteotomies was recorded. Restoration of the preexisting bone defects occurred in 83% of the patients. Three patients required repeat revision due to dislocation and one due to a postoperative periprosthetic fracture. None of the failures was attributed to the procedure itself.

**Conclusions:**

This new osteotomy technique may seem aggressive at first, but, at least in our hands, has effectively increased the speed of the femoral revision, particularly for the most difficult well-fixed components, but not at the expense of safety.

## Introduction

The transfemoral osteotomy described by Wagner [1] and the extended trochanteric osteotomy (ETO) popularized by Younger et al. [2] are the most commonly used in femoral revision, both when proximal or diaphyseal fixation of the new component has been decided [3,4]. These trochanteric osteotomies involve the creation of a longitudinal bone flap of various dimensions at the anterolateral femur, with the surrounding musculature remaining attached. They provide increased exposure of the fixation surfaces and adequate access to the canal for reaming and prosthesis insertion, but the overall complication rate has been reported as high as 24% [5]. Nonunion or proximal migration of the osteotomy fragment can occur [6-8], whereas the dorsal portion particularly of the proximal femur is vulnerable to intraoperative split fractures [3,4]. The latter can eventually compromise the potential for fixation of the revision stem [8].

We believe that such meticulous removal of the old prosthesis is not necessary, when distal fixation of the new component has been selected. Since proximal femur is to be bypassed, there is no reason either to be consumed in respecting it while exposing the interface surfaces or to jeopardize the new fixation with a distal fracture. We use instead a safe, straightforward transfemoral approach that minimizes operative time and effort for the removal of well-fixed stems without the danger of compromising distal fixation. It comprises of a transverse femoral osteotomy just below the stem tip and a premeditated segmentation of the remaining fixation areas which necessarily excludes the trochanteric region. With this approach, we have performed a series of femoral revisions with distally fixated stems, and no case of trochanteric migration or distal femoral fracture occurred.

### Surgical technique

The hips to be revised are templated to determine the appropriate stem length and the exact point of osteotomy. The revision stem requires at least a 4-cm depth of fixation in the intact distal diaphyseal bone. The osteotomy is performed just below the tip of the loose stem. The appropriate stem width may also be estimated, but is more accurately defined intraoperatively. To avoid a disruption of the vasto-gluteal sling and thus proximal migration of the greater trochanter (GT) postoperatively, we perform the procedure only through a modified posterolateral approach. This surgical approach has been already described [2]. It starts with the standard posterolateral incision, but it is centered over the tip of the greater trochanter and extends as far distally as needed to complete the osteotomy [2]. Once the hip joint has been exposed, an attempt to remove the prosthesis in a retrograde fashion is made. If this is not possible with standard techniques, we proceed to the proximal femur segmentation technique.

**Figure 1:**
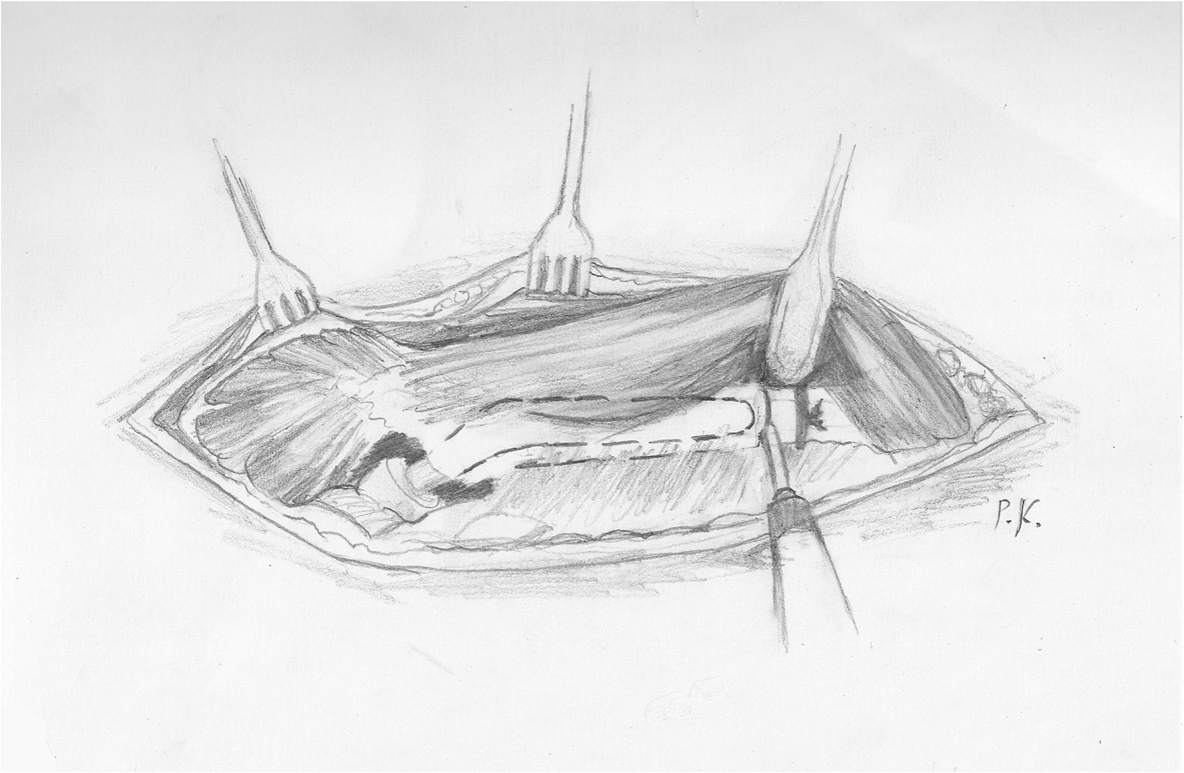
**The transverse distal osteotomy after prophylactic wiring is first performed.**

**Figure 2:**
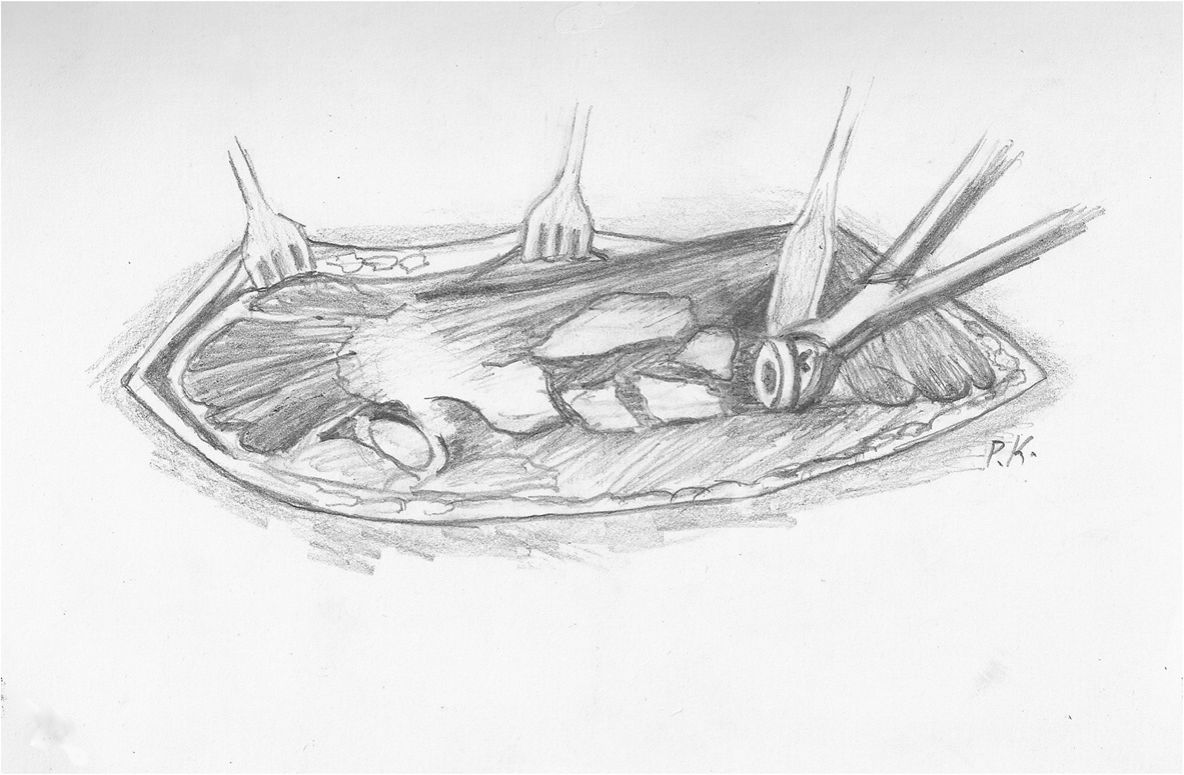
**Then controlled fractures of the proximal femur are generated with osteotomes.** They may extend from the osteotomy site distally to, as far as needed, proximally to facilitate the stem removal, but not closer than 2 cm from the vastus ridge. The continuity of the abductors with the vastus lateralis and the GT with the lesser trochanter is thus retained. Normally, this extent of fragmentation of the femur is enough for the removal of the old prosthesis, as the area of remaining fixation is usually distal and proximally the prosthesis is loose. If, however, there are still areas of proximal bone ingrowth or when a stable implant is revised, the posterior aspect of the intertrochanteric region can be violated to facilitate the stem removal, as long as the trochanteric continuity is retained anteriorly. After stem removal, the canal preparation and the revision prosthesis insertion are performed under direct vision.

**Figure 3:**
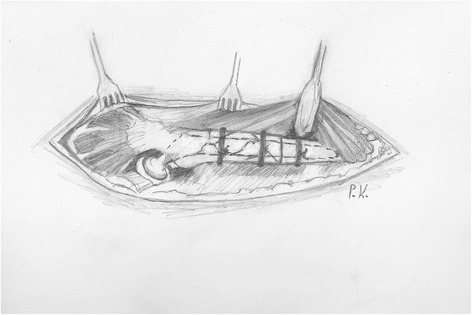
**The bone fragments are reattached to the new prosthesis with two or three cerclage wires, depending on the extent of the fragmentation.**

After the ligation of the perforating arteries, the vastus lateralis is separated from the femur along its posterior border and held with a Homann retractor, maintaining its origin on the vastus ridge. The predetermined osteotomy point is marked with multiple drills using a 4.5-mm drill bit. A prophylactic cerclage wire (1.2 mm in diameter) has been already placed 2 cm distally to the osteotomy point to prevent disruption of the intact distal femur. The osteotomy line is completed by connecting the drill sites with an osteotome or an oscillating saw (Figure 1). Then, multiple controlled fractures of the proximal femur to expose the fixation surfaces are generated with osteotomes of various diameters (Figure 2). The bone fragments are separated from the implant surface, but not from the attached soft tissues, by gentle lever movements of the osteotomes. This procedure starts just proximal to the osteotomy at the stem tip region and extends proximally up to 2 cm from the vastus ridge (Figure 3). A musculo-osseous-muscular sleeve comprising the abductors, the GT, and the vastus lateralis is, thus, left undisturbed. To further counteract the pull of the abductors to the proximal femur, the continuity of the GT with the lesser trochanter is retained, and the insertions of the vastus muscle group at the intertrochanteric line are preserved (Figure 2). After the removal of the old prosthesis, the canal is prepared under direct vision through the osteotomy. Sequential reaming of the canal can be performed, free of obstruction by the proximal femur anatomy and ensuring maximal canal fill for the predetermined length (Figure 2). The new prosthesis with the appropriate width is then inserted. The bone fragments are reattached with cerclage wires to the revision prosthesis, taking meticulous care to ensure that the wires do not touch the femoral component (Figure 3).

As far as the aftertreatment is concerned, we do not deviate from our standard hip revision mobilization protocol. One-third weight-bearing status is allowed, unless possible acetabular revision dictates otherwise, for the first 6 weeks and gradually progresses, as tolerated, to full weight-bearing by 12 weeks.

## Materials and methods

**Figure 4:**
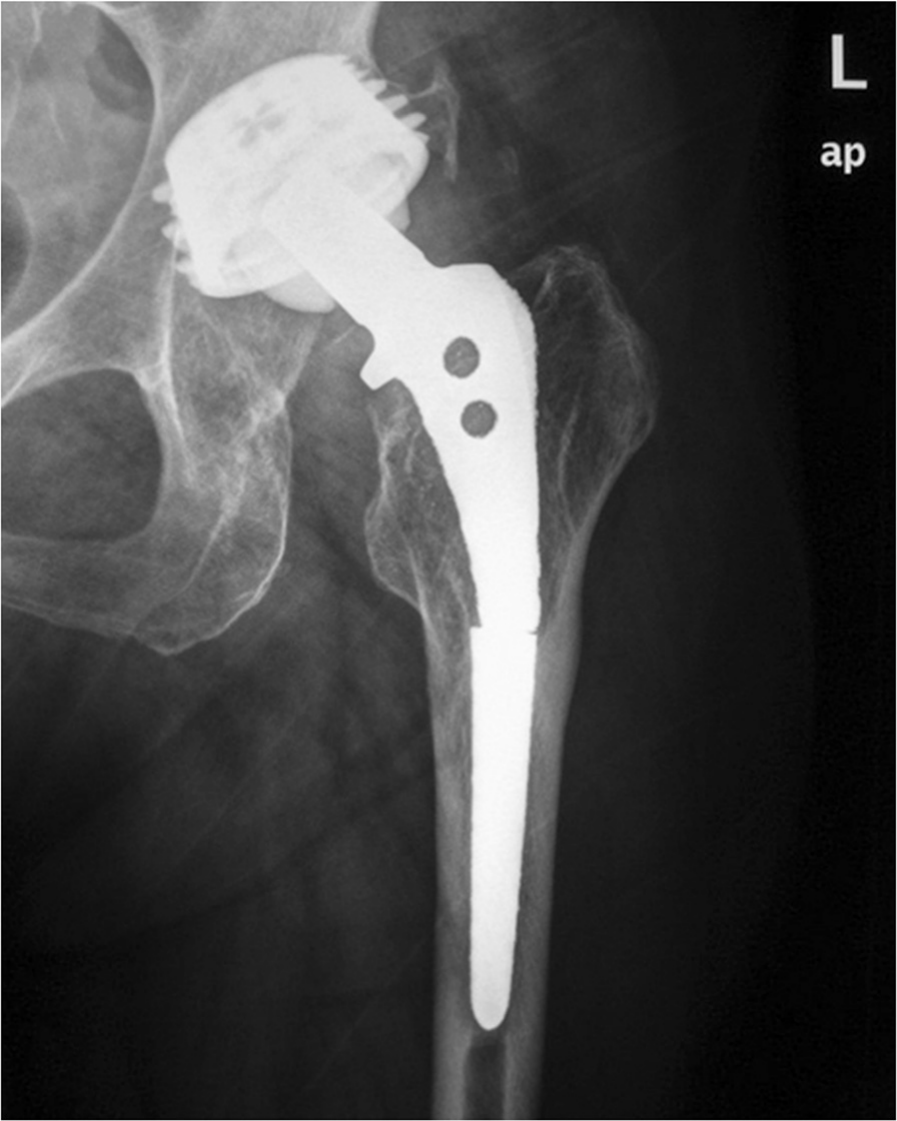
**A case of a broken stem with a well-fixed proximal part.** A 69-year-old female patient had an Autophor 900S stem implanted 15 years ago. This stem is entirely porous coated and has proximally two fenestrations for better anchoring. The arthroplasty became painful and an anteroposterior radiograph revealed a fatigue fracture of the middle of the stem. The distal part of the prosthesis seems firmly attached to the bone, whereas the stability of the proximal half is uncertain. During operation, this part could not be removed with standard techniques. Taking into account that distally the prosthesis is quadrilateral in cross section, it was decided to be revised with segmentation of the proximal femur.

**Figure 5:**
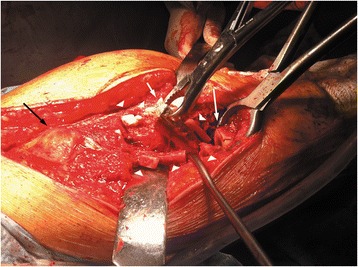
**Intraoperative photograph.** Note the bone fragments (arrowheads), which are separated from the implant surface, but not from the attached soft tissues, the extracted distal part (small white arrow), the transverse osteotomy (large white arrow), and the intact trochanteric region with the abductors and the vastus muscle group attached (black arrow).

**Figure 6:**
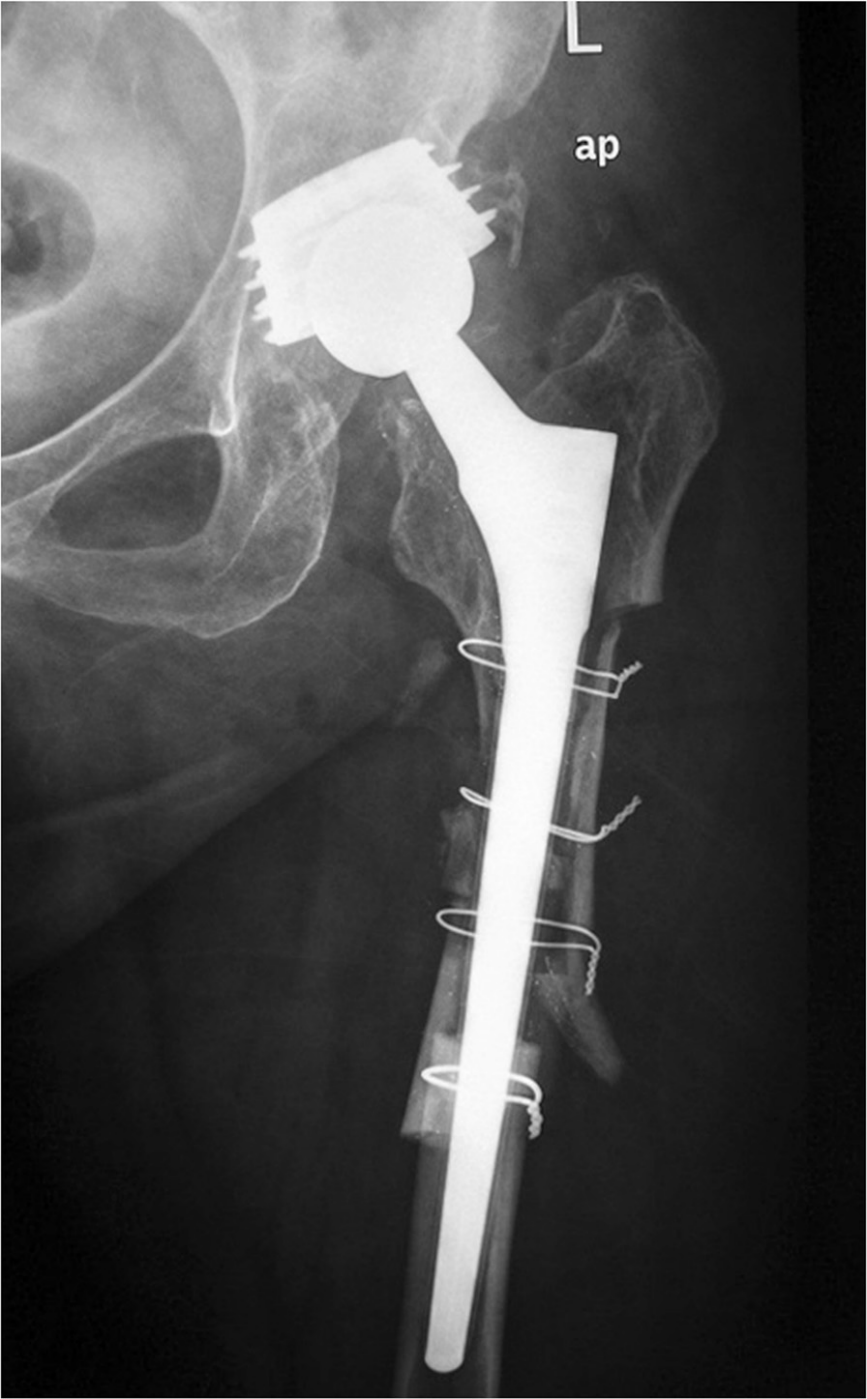
**The immediate postoperative radiograph.**

**Figure 7:**
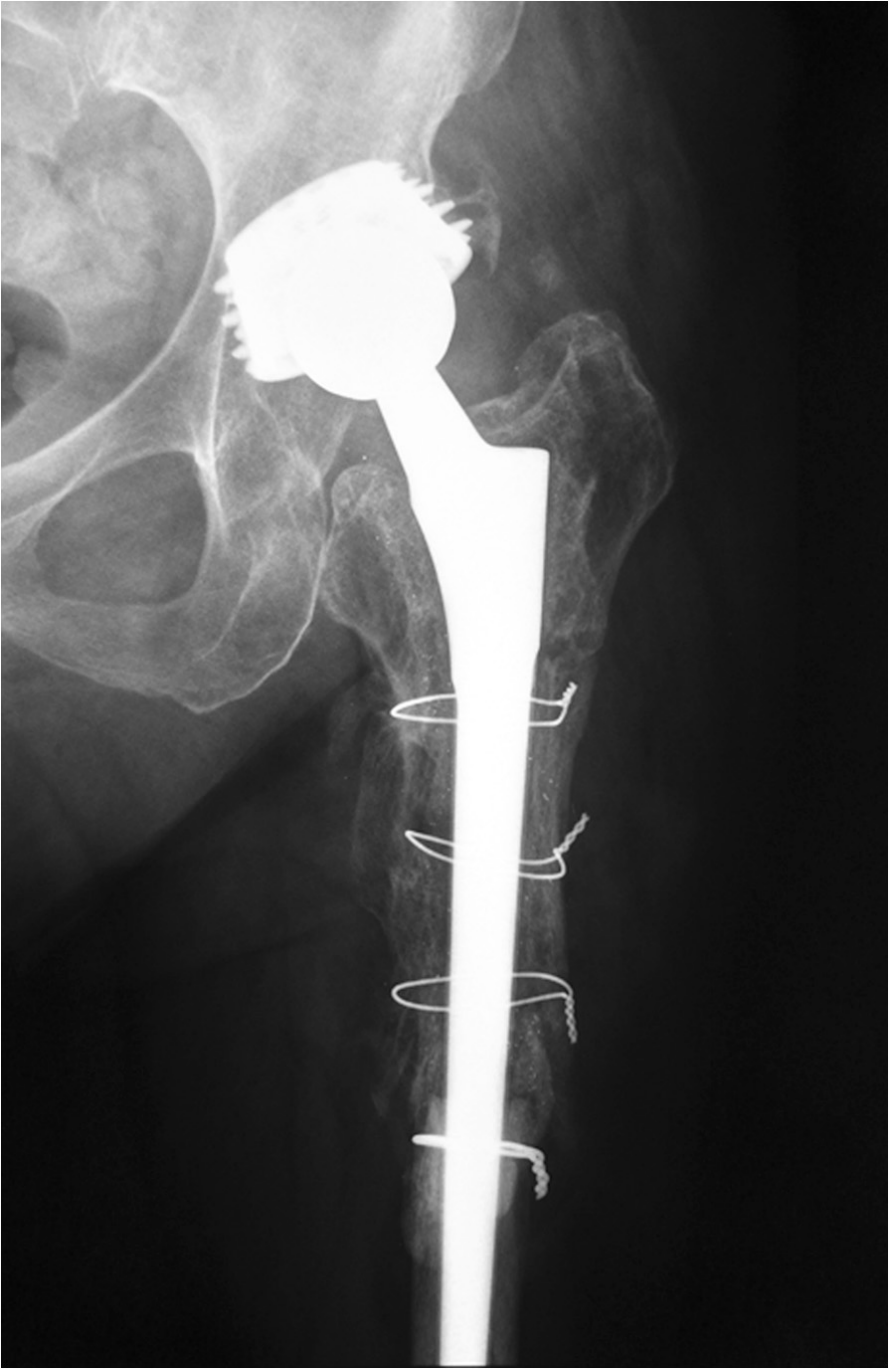
**Six months later, the fractures have healed, the osteotomy has united, and remodeling has occurred**.

We retrospectively reviewed the 47 consecutive patients who underwent the proximal femur segmentation technique by the first author in our tertiary university department between 2006 and 2012. They were 33 women and 14 men, with mean age of 68 years (range 39–88 years). The selection criteria included all the patients that required femoral osteotomy for stem revision due to aseptic or septic loosening and had adequate bone stock for diaphyseal fixation. After the consensus in our department, the inclusion criteria were extended to the patients with solidly fixed prostheses that are revised for reasons other than loosening (fractured or malpositioned stems, modular junction failures). Exclusion criteria were Valle and Paprosky [9] type 4 bone defects that precluded a solid distal fixation. Those cases with easily extractable, without trochanteric osteotomy, prostheses during operation were also excluded. Broken stems with loosened proximal parts that can be managed successfully with trephining of the retained distal segment fall into this category. However, the proximal part cannot always be easily removed (Figures 4, 5, 6, and 7).

For the 47 patients, the reasons for revision are shown in Table 1. In total, 12 patients (26%) had a stable prosthesis and 35 (74%) a loose one. Their pre-revision bone defects are demonstrated in Table 2. For 36 patients, this was the first revision, for nine the second, and for two the third. The original arthroplasty was uncemented in 40 patients and cemented in seven, whereas the time from the first arthroplasty to the index revision was on average 9.4 years (range 2–18 years). In 30 of these revisions, the femur alone was revised. In the remaining 17 patients, the acetabulum was also revised. For the latter dual-component revisions, cell salvage was performed during the procedure. The electronic database was searched and the number of blood units transfused in each case was recorded. The Wagner stem (Wagner SL Revision® Stem, Zimmer Warsaw, IN, USA) was used in 40 cases, while the rest (seven patients) received a newer modular and curved, fluted, tapered stem (Profemur® R stem, Wright Medical Technology Inc., Arlington, TN, USA). Due to the spontaneous regeneration of the proximal bone stock seen with these revision stems [3], strut allografts were reserved only for the younger patients with Valle and Paprosky [9] type 3 defects (three patients, mean age 49 years, range 39–55 years).

**Table 1:** **The reasons for revision for our study group**

**Indications for revision**	***n***	**%**
Aseptic loosening	27	58
Stable prostheses	12	26
Recurrent dislocation	8	17
Broken stem	2	4
Modular neck fracture	2	4
Periprosthetic fracture	6	13
Vancouver B2	4	9
Vancouver B3	2	4
Periprosthetic infection^a^	2	4
Total	47	100

^a^Second-stage reimplantation after a Girdlestone resection.

**Table 2:** **Distribution of pre-revision bone defects**

**Pre-revision defects** ^**a**^	***n***	**%**
Type 1	6	13
Type 2	11	24
Type 3	21	44
A	18	38
B	3	6
No bone loss	9	19
Total	47	100

^a^According to the system of Valle and Paprosky [15].

Patient reevaluation was done at regular intervals (first, third, and sixth months and every year thereafter) and included clinical assessment with the Harris Hip Score (HHS) and standardized digital radiography of the operated hips. Migration was assessed by measuring the vertical stem subsidence according to the method of Callaghan et al. [6], as a change in the vertical distance from the proximal tip of the greater trochanter to the shoulder of the stem. A computer-assisted method was used to perform these measurements. The radiographs were originally DICOM and transformed into TIFF grayscale format without compression. These digital images were processed via Roman v1.7 software (Roman software version V1.70; Robert Jones and Agnes Hunt Orthopaedic Hospital, Oswestry, UK; http://www.cookedbits.co.uk/roman/). A difference of 5 mm or more in the vertical direction between the immediate postoperative measurement and the measurement at follow-up was considered to indicate vertical subsidence. Proximal bone restoration was evaluated according to the method by Böhm and Bischel [3]. Cases were classified as A (increasing defects), B (constant defects), or C (osseous restoration).

## Results

During surgery, no fracture of the trochanters or the distal femur occurred. Mean duration of operation was 102 min (range 80–189 min) for femoral and 174 min (range 122–235 min) for acetabular-femoral revisions. Overall, 27/47 of the patients (58%) received blood transfusions, 13/30 (43%) in femoral component and 14/17 (82%) in dual-component revisions. For each transfused patient, 2.3 blood units were used on average (range 1–8), with 91% requiring less than 4 units.

Mean follow-up was 28 months (range 6–70 months). Two patients were lost to follow-up and one died for reasons irrelevant with the operation. The mean preoperative HHS was 31 (range 1–80), which improved to a mean of 83 (range 48–96) at the final follow-up (*p* < 0.001). At this evaluation, all the stems were radiographically stable. No case of GT proximal migration was observed. All the osteotomies united reliably between 3–9 months (mean 5.2 months). A mean subsidence of 2.4 mm (range 0.8 to 4.2 mm) was measured at the latest follow-up, and no case of rapid postoperative stem migration was recorded. The most recent radiographs showed advanced remodeling of the osteotomy and the fractured areas in 42/44 patients (95%). Restoration of the preexisting bone defects was seen in 29/35 patients (83%), no change in 4/35 patients (11%), and increasing bone defects in only 2/35 hips (6%). There was complete incorporation of the bone graft whenever it was used.

Complications were recorded in six cases. None was attributed to the osteotomy technique. Two patients presented a superficial wound infection that resolved after surgical debridement and antibiotic treatment. Four revision stems required repeat revision. Two patients were reoperated in the immediate postoperative period due to dislocation. In the first case, the stem was undersized and was revised to a longer one, without dislocation recurrence. In the second case, a high angle of anteversion of both the stem and the cup was corrected. Another patient, with Paprosky type 3B pre-revision bone defects, had a late recurrent dislocation without major subsidence. During operation, 5 years after the first revision, a retroverted stem was found. An attempt to use a longer Wagner component in the distal third of the femur, in mostly cancellous bone, did not provide a safe fixation, even with the largest available stem diameter. A proximal femoral replacement prosthesis was eventually used for the femoral reconstruction. The same component received another patient who sustained a Vancouver B3 periprosthetic fracture after a fall 3 years postoperatively and had defects classified as type 4.

## Discussion

The transfemoral approach [1] and the ETO [2] facilitate femoral canal exposure, while preserving the hip abductors and vastus lateralis musculature in continuity. The vastus is thought to counteract the pull of the abductors in the coronal plane, thus avoiding proximal migration and promoting osteotomy union [10]. However, the reported incidence of proximal migration of the ETO fragment is up to 6.6% [4,8] and the incidence of nonunion similarly up to 11% [4,7]. Another important consideration that must be taken into account, when using these trochanteric osteotomies, is that when there is an obvious curvature in the frontal and/or sagittal planes, these osteotomies cannot provide a straight-shot access to the canal, thus increasing the risk of intraoperative perforations of the femoral shaft [7,8]. Even more commonly, intraoperative split fractures of the paper-thin, proximal cortices during implant and cement removal can occur. Although the osteotomized anterolateral bone flap is folded back, the contralateral interface surfaces remain unexposed. Despite the use of modern removal instruments [8], the rate of proximal femur fractures has been reported to be between 4% [4,8] and 60% [3]. What is more important is that these fractures concern the more distal portion of the proximal femur, as this is the usual area of remaining fixation [8]. Furthermore, they are uncontrollable, because there is nothing to stop them extend peripherally [3,8]. In many cases, the attempt to remove a well-fixed prosthesis results in further loss of proximal bone stock and an uncontrolled fragmentation of the distal femur, which usually necessitates a longer revision stem.

We propose an alternative to these trochanteric osteotomies, when anything else than a completely loose implant is anticipated. It comprises of a transverse femoral osteotomy just below the stem tip and a subsequent preplanned segmentation of the proximal femur to quickly remove the old prosthesis. It combines the advantages of an extremely wide exposure of component fixation surfaces with the preservation of soft tissue attachments to cut bone. The technique is based on the fact that proximal femur fractures do not interfere with the ultimate stability of a distally anchored component, as long as the hip abductor mechanism remains efficient. For this purpose, the GT is kept intact and the continuity of the abductors to the quadriceps is retained to avoid proximal trochanter migration. The fragmentation extends up to, but excludes, the vastus lateralis origin. To further minimize the possibility of GT migration, its continuity with the lesser trochanter is preserved at least to one femoral cortex. On the other hand, the transverse osteotomy serves primarily to protect from the distal extension of the fractures; it also, however, allows the surgeon to correct any proximal deformity and gain a straight trajectory down a bowed femur. Through the osteotomy, the canal machining and the intimacy of the implant-bone contact can be unimpededly checked under direct vision. We attribute the abolition, despite the normal weight-bearing protocol, of gross subsidence seen with tapered, fluted stems [3] and the absence of intraoperative distal fractures in our series to this appropriate femoral preparation and accurate sizing. Altogether, the speed of stem removal and reimplantation allowed us to reduce the total operation time in relation to other revision techniques (102 min (range 80–189 min) for femoral and 174 min (range 122–235 min) for acetabular-femoral revisions vs 185 min (range 120–330 min) for femoral and 225 min (range 75–470 min) for acetabular-femoral revisions with ETO [11]), and this with minimal overall damage to the proximal bone stock, as no attempt to disrupt fixation surfaces with tools such as power burrs or drills is made. On the contrary, not only the bone fragments are reattached with cerclage wires to the new prosthesis and the proximal femur is reconstituted, but also florid bone remodeling and restoration of the preexisting bone defects is observed, presumably due to the fracture healing response and/or the favorable biomechanics of the cementless fluted, tapered implant-bone interface [3].

This feature of proximal bone reconstitution was behind our decision to use the proximal femur segmentation technique also for revision of solidly bonded stems. Among various techniques described in the literature [11,12], a two-stage removal is invariably advocated for these revisions: after ETO, the stem is sectioned with burrs between the proximal and the distal tapered part [11-13]. Then, the proximal segment is removed using tools such as Gigli saws and burrs, and the distal is trephined with reamers 0.5 mm larger than the diameter of the distal segment [11-13]. Our rationale against this approach is that to the shortcomings of the ETO those related with trephining are added. These include not only the intraoperative risks of femoral perforation and trephine breakage [13-15] but also the thermal damage to the cortex, which can extend to a greater area [13,15,16]. These dangers are exacerbated with older non-tapered cylindrical designs, which due to large distal dimensions require the use of larger reamers [14]. In these cases, since the trephines are cylindrical in shape, but also with the newer tapered but rectangular self-locking cross sections, unnecessary removal of the host bone takes place and the theoretical advantages of bone preservation of trephining are eliminated [15]. In any case, the disruption of completely ingrown surfaces with tools at the proximal implant has a high rate of iatrogenic fragmentation of the femur, which irreversibly compromises the potential of the revision prosthesis for fixation, either proximal or distal. In many cases, this attempt results in frail primary stem stability, which ultimately requires a re-revision with all relevant risks and complications. We consider the results of our approach more predictable, and although the method appears initially to be destructive of uninvolved bone, proximal bone reconstitution is eventually achieved. Only for the broken stems we favor trephining over the segmentation technique. In these cases, the proximal part is usually loose and can be easily removed without the need for disruption of fixation surfaces. The risks of distal part trephining, however, remain.

The major limitation of the proximal femur segmentation technique is that it can only be performed in combination with a posterior approach, as, to avoid a postoperative proximal trochanteric migration, the integrity of the vasto-gluteal sling is a conditio sine qua non. Furthermore, it cannot be combined with proximally coated revision implants. When reconstruction of the deficient bone stock is selected and press-fit fixation of the new prosthesis is intended, the proximal femur should be circumferentially kept intact and the older osteotomies appear to remain the only viable option. Another consideration is that we have used so far this technique only with tapered, fluted, grit-blasted stems. Although we do not have the relevant experience, we believe that it can be also combined successfully with other distally fixated revision systems, such as the extensively porous-coated cylindrical stems [17]. The most frequent postoperative complication encountered was dislocation. All of them, however, were due to initially undersized or malpositioned stems and not due to subsidence. As these failures were recorded early in our series, we attribute them to the learning curve in implanting this system. As far as the blood loss is concerned, although the available data lacked important confounders such as the preoperative hemoglobin levels, our results (43% rate of transfusion in femoral and 82% in dual-component revisions, 2.3 blood units for each transfused patient on average) appear to be consistent with the literature [18,19], despite the proximal femur segmentation. Mahadevan et al. [18], after studying the records from 146 revision total hip replacements (THRs), found a transfusion rate of 42% in femoral component and 73% in dual-component revision, but each transfused patient received 3.5 blood units on average. Similarly, Sharma et al. [19] found transfusion requirements of 2.5–5.2 units for each transfused patient after revision THR, depending on the preoperative hemoglobin levels. Our practice is to cross-match 4 blood units before revisions with this technique, which have been proved enough in the majority of the cases.

## Conclusion

The proximal femur segmentation technique facilitates the removal of the old prosthesis and the implantation of the revision component, even when proximal femoral bone deformity is present. With the steep increase in the prevalence of failed total hip arthroplasties, the speed and safety of this technique may be proved invaluable to the femoral revision surgery.
